# Supplementary material for: Four New Species of Small-Spored Alternaria Isolated from Solanum tuberosum and S. lycopersicum in China
Source: J Fungi (Basel). 2023 Aug 27;9(9):880. doi: 10.3390/jof9090880 (PMC10532295; doi:10.3390/jof9090880)
Supplement: Supplementary file 1 [file jof-09-00880-s001.zip › jof-2577294-supplementary.pdf]

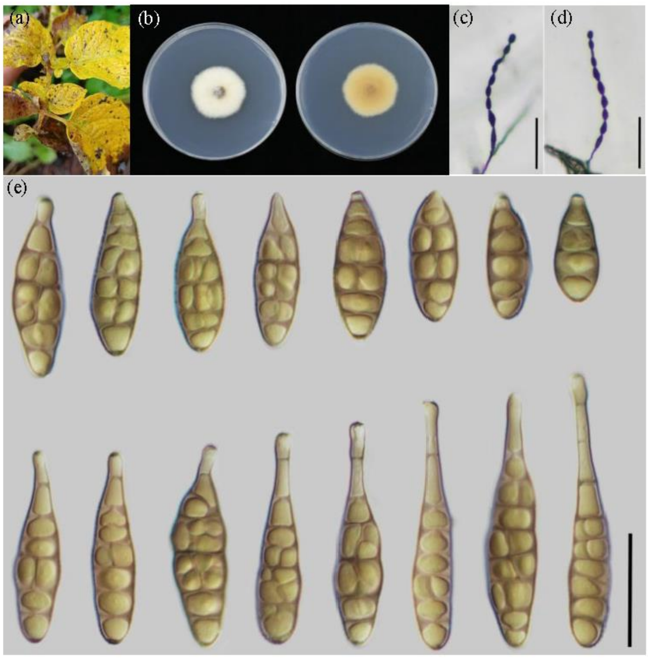

`

Figure S1. Morphology of *A. gossypina*: (a) Diseased leaves in field; (b) Colony phenotypes (on PDA for 7 days at 25 °C); (c, d) Sporulation patterns; (e) Conidia (on PCA at 22 °C). Bars: c–d= 100 μm; e = 25 μm.

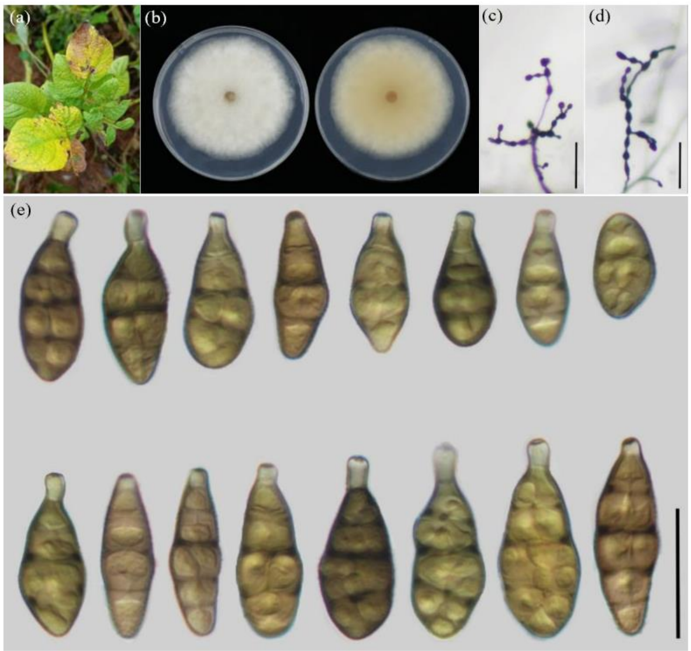

Figure S2. Morphology of *A. arborescens*: (**a**) Diseased leaves in field; (**b**) Colony phenotypes (on PDA for 7 days at 25 °C); (**c, d**) Sporulation patterns; (**e**) Conidia (on PCA at 22 °C). Bars: c–d= 100 μm; e = 25 μm.
